# Supplementary figures and images for: Single versus dual-rate learning when exposed to Coriolis forces during reaching movements
Source: PLoS One. 2020 Oct 19;15(10):e0240666. doi: 10.1371/journal.pone.0240666 (PMC7571717; doi:10.1371/journal.pone.0240666)

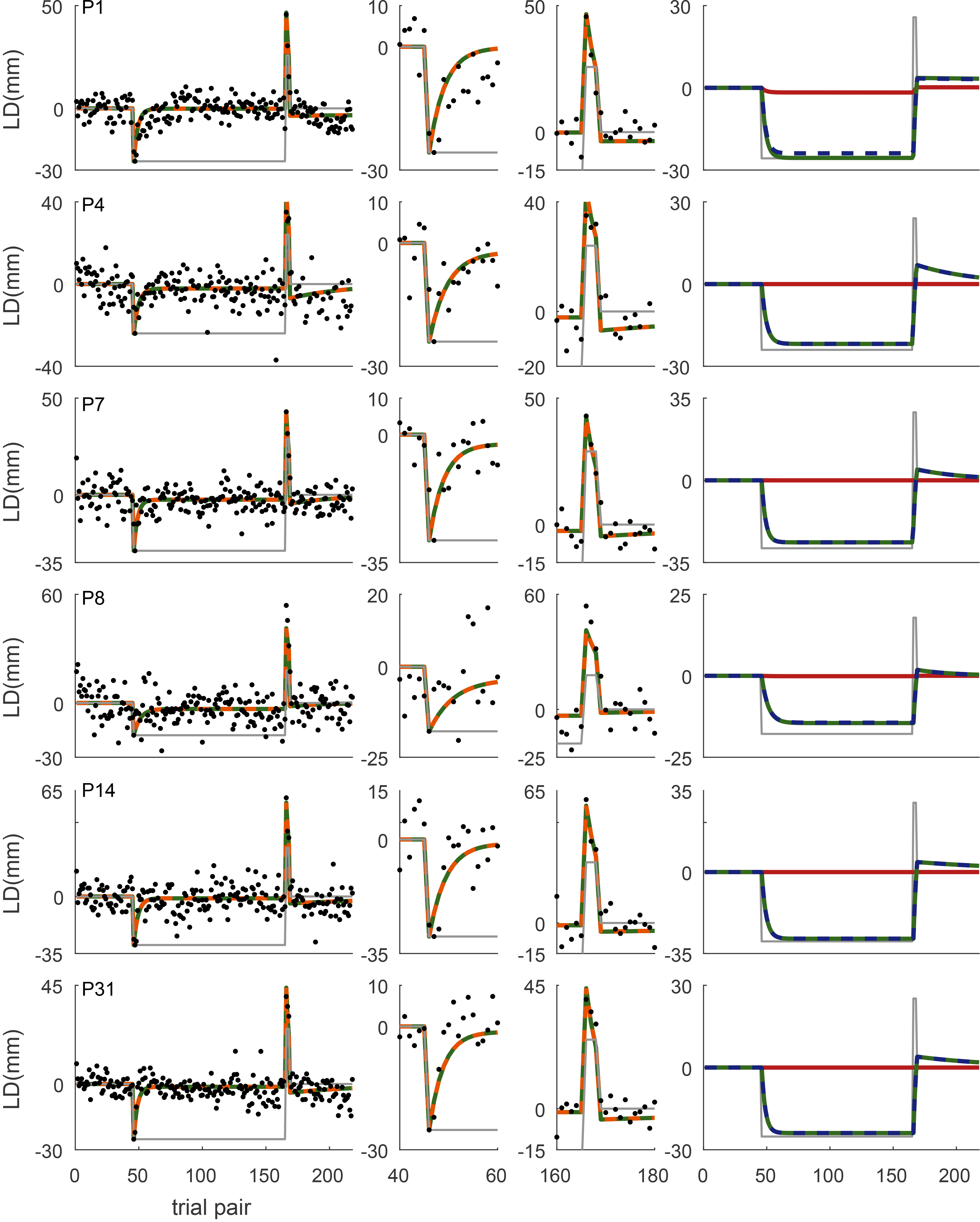

Supplement: S1 Fig — Same legend as in Fig 4. Dual-rate model fits predicting the same pattern as the single rate model fits by setting one of the two states to zero. (TIF) [file pone.0240666.s001.tif]

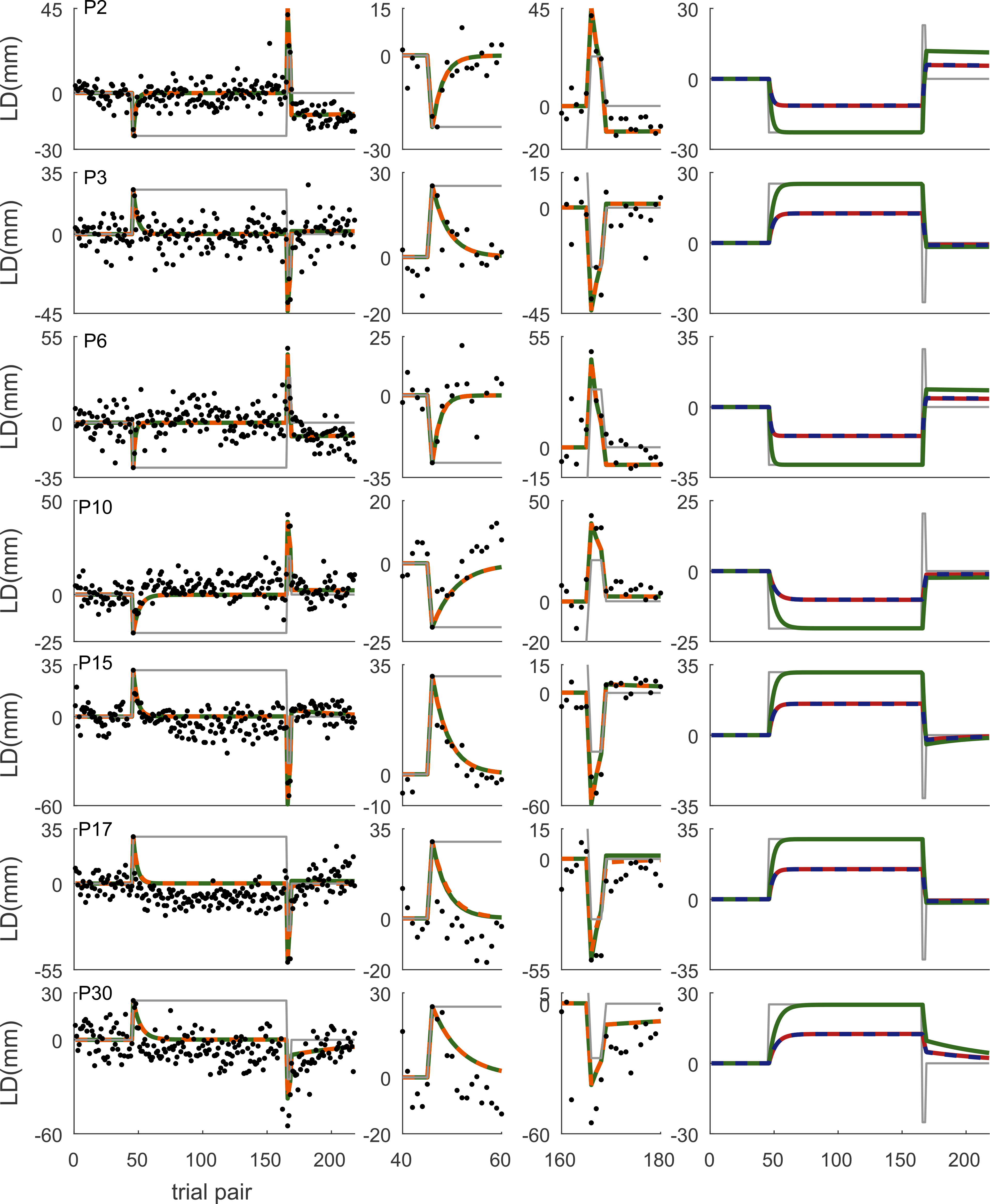

Supplement: S2 Fig — Same legend as in Fig 4. Dual-rate model fits predicting the same patterns as the single rate models fit by setting both states equal. (TIF) [file pone.0240666.s002.tif]

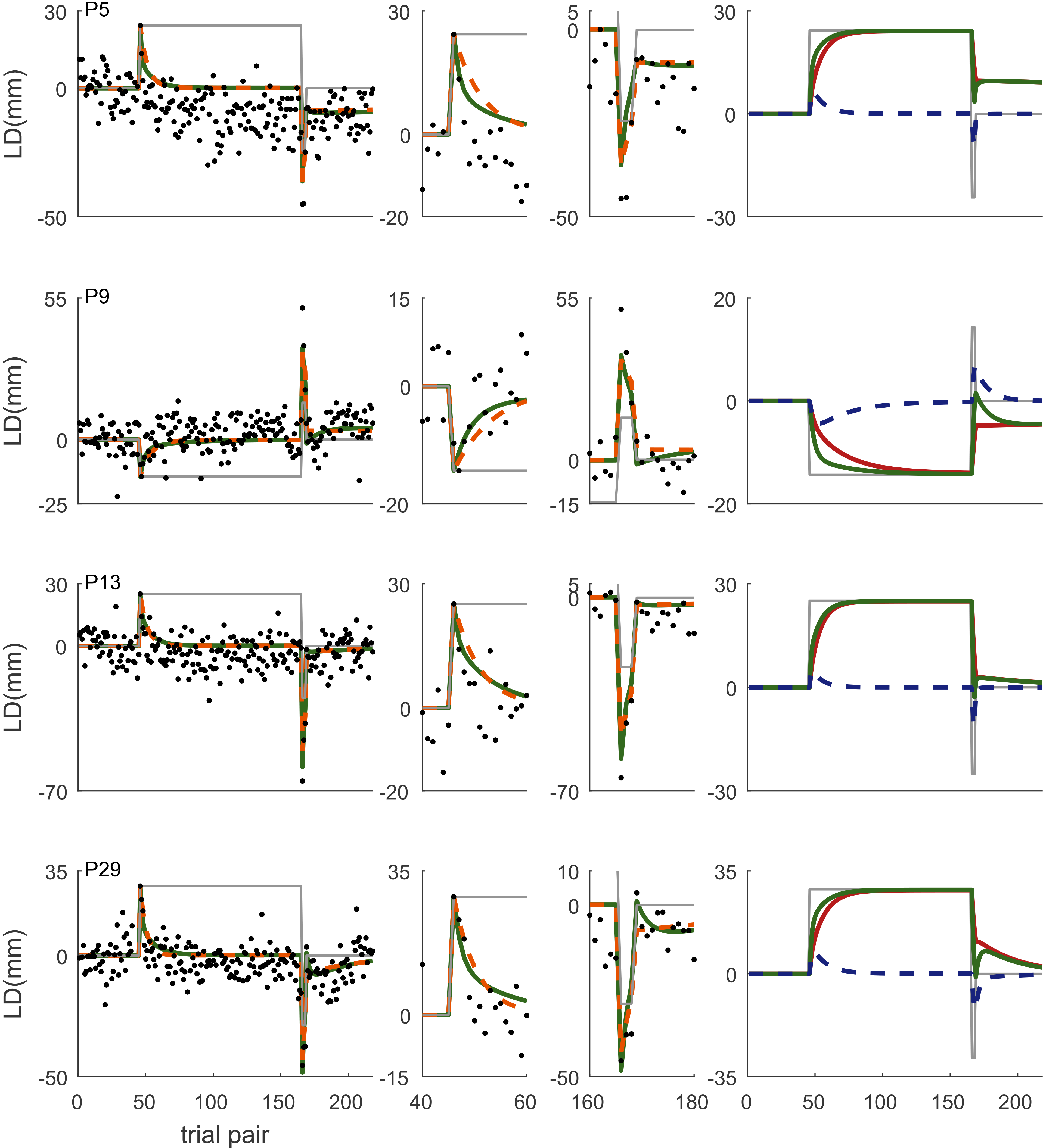

Supplement: S3 Fig — Same legend as in Fig 4. Model fits of the dual-rate model showing typical dual-rate pattern. (TIF) [file pone.0240666.s003.tif]
