# Supplementary material for: Single versus dual-rate learning when exposed to Coriolis forces during reaching movements
Source: PLoS One. 2020 Oct 19;15(10):e0240666. doi: 10.1371/journal.pone.0240666 (PMC7571717; doi:10.1371/journal.pone.0240666)
Supplement: Attachment — Submitted filename: Response to Reviewers.docx [file pone.0240666.s004.docx]

*Revision PONE-D-20-15875 “Single versus Dual-Rate Learning when Exposed to Coriolis Forces during Reaching Movements” by Rudolph et al.*

*We thank the reviewers for their evaluations and insightful comments. We hope to have dealt with them appropriately, and now present an improved manuscript. Below, you will find the reviewers’ comments, and our reply in italics.*

Reviewer #1: The authors investigated whether adaptation to Coriolis forces is governed by a single or dual-rate adaptation process. To do so, they let participants perform a pointing task in a room that could rotate either clockwise or counterclockwise, introducing Coriolis forces on the arm. The manuscript reads as very careful work in which much attention has been paid to the design of the experiment and to parts of the statistical analysis. Yet, for me as someone who studied the dual-rate process, it leaves two pressing questions unanswered. First: did the authors have enough power to make a between-groups comparison of dual-rate model fits with two additional parameters? Second, and related: recent work by Albert & Shadmehr 2018 (Journal of Neurophysiology) has shown statistical problems with fitting the dual rate model. To what extent did the procedure used by the authors fix these problems? As I believe that these issues need to be addressed before being able to interpret the data as evidence that adaptation to Coriolis forces is governed by a single-rate process, I have suggested a major revision.

*We appreciate these points, of which the first – sample size – we will address below. Regarding the second on fitting the dual-rate model: we are very aware of the issues raised by Albert and Shadmehr (2018). They compared a least-squares algorithm (LMSE, similar to ours) and an expectation maximization (EM) algorithm to recover the hidden states of simulated data in four paradigms. They considered the following paradigms: abrupt perturbations that included or excluded error clamp trials (paradigm 1 and 2), a paradigm with a gradual perturbation (paradigm 3), and a paradigm that demonstrated spontaneous recovery and decay of motor memory due to set breaks (paradigm 4; similar to our paradigm).*

*While the EM algorithm recovered the hidden states better in the first three paradigms, both algorithms (EM and LMSE) converge equally well on the correct hidden states when applied to the spontaneous recovery paradigm (paradigm 4, see their Figure 3, fourth column). Hence, their extensive analysis does not argue against our fitting approach.*

Major comments

Sample size

In my experience, adaptation is highly variable between participants. Often, I enthusiastically presented the data from 9 participants at a conference, having to change my conclusions when more participants were tested. The sample size of 10 participants in the control group and 17 participants in the experimental group seems quite small to me. The standard errors in Figure 2 suggest that participants within a group behaved relatively consistently, which is re-assuring. However, I’d like to see more evidence that the data were normally distributed around the mean. This is important for the interpretation of the adaptation curves presented in figure 3.

*Our sample size was quite similar to sample sizes in previous studies (e.g. Smith et al. 2006; Ingram et al., 2011; Threwartha et al., 2014). But more importantly, we performed our analysis at the single subject level. We provide data and fits of each participant (see original Supplement) and in none we found supportive evidence for the two-state model. Indeed, at the group level, the small standard errors in Figure 2 further support this claim. To meet the reviewer’s request, below we provide an overview of the distribution of errors across participants as a function of trial number in the experiment, showing that data are normally distributed.*

Robustness of model fits

I am also concerned about the robustness of the model fits. I couldn’t find information on the quality of the fits: confidence intervals on the parameters in table 1, for instance. I never worked with the Bayesian Information Criterion, but if both models fitted the data poorly due to a high level of noise, wouldn’t the model with fewer parameters win? In that case, the finding that the BIC was higher for the single-rate model could be an artifact of the data being noisy. Without more evidence on the statistical reliability of the model fits, adaptation to Coriolis forces could also be governed by a dual rate process with a very slow ‘slow process’, I think. Of course, a fundamental question is when the slow process is so slow it can be disregarded.

*We have now provided more information about the goodness-of-fit, by adding the R-squared of the fits. Note that R-squared is theoretically only valid for a linear model fit, and problematic for a non-linear model like the single- and dual-rate learning models used here.*

*This is also the reason that we restricted our computation of R-squared to the fit and data of the second learning (B) block and spontaneous recovery block. These values range between 0.32 and 0.80, with a mean of 0.64 and SD of 0.12 (across both models and all participants).Adding the baseline and block A mainly adds variance that cannot be captured by the deterministic models. Second, the reviewer is correct that high levels of noise and poor fits may work against the dual-rate model. However, in the majority of our participants the dual-rate model converges to a single-rate model, indicating that a dual-rate process is an inappropriate description of the data.*

Potential bias

The text mentions that the data converge to zero in Figure 3. Looking at Figure 3, the data do not seem to converge towards zero. For CW-CCW they do, but not for CCW-CW. If there is a bias in the data with either leftward or rightward errors being more likely, this could have affected the results in the washout phase.

*We assume that the reviewer is referring to the asymptote of the error in Block A, where the participant is exposed to the first rotation direction for an extended period of time. Indeed, the error deviates slightly from zero at the end of this exposure, indicating that the participants are overcompensating for the external forces. However, this would work in favor of showing spontaneous recovery if the learning process is based on multiple states (which we didn’t see), as it is an even stronger ‘signature’ of the behaviour learned in this block. We adapted the manuscript as follows:*

*“At the start of block A, the LD initially clearly deviates from zero, but then quickly returns to zero or even slightly crosses zero (CCW-CW group) towards a level opposite to the initial deviation (indicating overcompensation) in ∼10 trial pairs.”*

Minor

Line 25: I would phrase the sentence a little bit differently. Many types of errors contribute to motor adaptation (e.g. target errors, performance errors, sensory prediction errors). All these errors reflect ‘mistakes’.

*Done. We have rewritten this into: “In motor learning, one of the types of mistakes is the difference between the actually sensed and the internally predicted sensory consequences of a movement.“*

line 86; please mention how many participants were excluded due to failure to follow task instructions and how they failed to follow the task instructions

*Done. We have provided further details: “Data of seven participants were excluded from analyses due to technical problems (five participants) or failure to follow task instructions (moving their arm while the room accelerated or during block B)”*

Line 131: why were these movement times chosen?

*These were based on pilot experiments. Coriolis forces depend on the movement velocity of the arm; the higher the velocity, the larger the experienced forces. In pilot versions of the experiment, and experience with similar experiments on a robotic interface, we found that this is a comfortable movement time when we push the participant for speed.*

Line 162: how was it determined whether more practice trials were needed?

*The familiarization phase lasted until participants were able to comfortably make reaches at the required speed.” We clarified the text:*

*“Prior to the main experiment, participants were familiarized with the task in at least 90 trials, training them on the movement timings and on reaching under the use of shutter glasses, up until they were comfortable with the task.”.*

Line 201: Why was a cartesian coordinate system used? Compensating for a rotation would suggest using a spherical coordinate system to me. That way the lateral error could be expressed in degrees azimuth

*Lateral deviation is a commonly used measure in force field adaptation and was also used in the seminal paper by Lackner and Dizio in 1994. We could have opted for a spherical coordinate systems, but this is just a re-parametrization of the data, and more difficult to interpret here.*

205 Does the y-position refer to the sagittal position? The y-postition can refer to multiple axes

*Done. We have clarified the text as follows:*

*“The y-axis was pointing forward, parallel to the mid-sagittal plane of the participant, the x-axis pointing rightward, and the z-axis pointing up from the screen.”*

*We have also added a depiction of the axis system to Figure 1C.*

The LD abbreviation is unnecessary

*We are not sure whether the reviewer is referring to a specific use of LD or that we should have written ‘lateral deviation’ throughout the manuscript. It is very common in the literature to abbreviate error measures. We prefer to keep it, as we use it throughout the manuscript, but have removed it from the figure and section titles.*

Line 241: please mention that the fit procedure is explained later

*We have added the following sentence:*

*“We will first explain the details of the two models, followed by the fitting procedure and statistical methods to compare the two models”*

Line 336: It isn’t clear whether the washout block or the model fits are the crucial part of the data analysis

*Both the behaviour in the washout block and the model fits contribute to answering our question whether adaptation to Coriolis force is a single- or multi state learning process. We have rewritten the sentence as follows:*

*“We tested the emergence of spontaneous recovery effects during this washout period, to examine whether reach adaptation to Coriolis forces is best described by a single-rate or dual-rate learning process using a model-based analysis.”*

Figure 2. Having the figures and text separately, Figure 2 was difficult to grasp with all the different colours.

*Point taken, but in the published manuscript, the figure and text will be close together alleviating this concern.*

Line 402: spontaneous recovery of the adaptation to perturbation A?

*We presume that the referee want us to amend the sentence. We now write:*

*“Alternatively, if there are no signs of spontaneous recovery of the adaptation to the perturbation experienced in block A in the washout block, this hints at a single-rate learning process.”*

Figure 3: The data in block B are very difficult to see. Where is the SE?

*We agree that the SE doesn’t show very clearly in block B. Part of this is because the SE in the vector graph was obscured when converted to the submitted PDF, but it should also be noted that the SE is small.*

Line 430: This sentence is difficult to understand. What do the authors refer to?

*We thank the referee for spotting this writing error! Obviously we are looking here at the after-effect of block A in the washout phase. We have rectified the sentence as follows:*

*“The aftereffects of block A quickly fade away in about 10 trial pairs …”*

Line 439: Please specify what the typical pattern would be

*Done. We now write: “However, even though the observed aftereffect (magenta, top-right panel) has the same sign as the aftereffect of the corresponding control group (blue, top-right panel), it does not show the pattern typically observed in spontaneous recovery. That is, we do not observe that the compensation to the initial perturbation quickly rises (due to the forgetting of the fast process) up to maximal recovery and then slowly decays due to forgetting of the slower process.”*

Line 443: Please present the data such that this can be seen in the figure

*Done. We have added the baseline performance and fluctuations to the right of trial pair 218 as well as some other small amendments to Figure 2.*

Figure 4: It would help me to provide column titles in the figure

*Done.*

Reviewer #2: I commend the authors on an original and well-designed study that is novel and relevant to the field. The manuscript presents a study in which participants perform a reaching task while being exposed to Coriolis forces. The adaptation process is described in the different phases of the experiment and two models are proposed to explain the learning process: a single rate and a dual rate model, of which the single rate model seems to have the best fit. The authors do well in highlighting some limitations to the study and to discuss the meaning of these results in light of previous studies. Overall, the manuscript is of good quality, but some components of the methods and results section lack clarity in their reporting.

Major issues

I feel some information is presented in an order that breaks up the flow of the manuscript. A statement of the main research question seems to be subtly mentioned in line 61-65 without stating any hypotheses or operationalization. After this the introduction is finished with a summary of the methods and the results section. An operationalization of the research question does seem to be mentioned in the results, with the paragraphs on line 386-408. I believe it would improve the readability of the manuscript if this part of the results would be moved to the introduction and that the introduction would end with a clear statement of the aims or research question.

*Done. We have operationalized our research question in the introduction as follows:*

*Line 62: “ Here we ask the question whether this rapid adaptation is mediated by a single-rate process, analogously to the rapid adaptation to hammer dynamics, or governed by a multi-rate learning process.”,*

*And on line 72, we added: “We hypothesized that if the adaptation was governed by a multi-rate learning process, reach compensation for the first rotation (spontaneous recovery) would be observed during this phase”*

Minor issues

1. Line 108 (Fig 1.). The grey area in this figure is hard to see (both printed and on screen). Please use a darker shade of grey.

*Done.*

2. Line 121-122. On the next page it is introduced that this statement is only true for about half the trials. Please report both procedures as to the opening and closing of the glasses here.

*We do not quite understand this remark because this sentence captures the procedures for both opening and closing of the shutter glasses.*

3. Line 125. The figure caption for Fig 1C mentions that vision is occluded during the reaches, but this sentence indicates participants need to locate the target prior to the forward movement when shutters are closed. Please clarify when shutters were closed.

*Done. We amended the text as follows:*

*We removed “when the shutter were closed” from line 127, to emphasize the role of touch in localizing the target closest to the body.*

*We added a sentence on line 198: “This meant that the shutters were only opened when the participants index finger was at the start position, which could be located by a tactile cue (see above).”*

4. Line 131-136 describe the required timing of the reaching movement. What happened with trials that were not inside the time limit? Were these removed? Was there any procedure in place in case participants were fast or slow repeatedly?

*All trials were included, as all affect learning. We added the following sentence to the revised manuscript: “Because every movement contributes to the learning, irrespective of their durations, we did not reject trials based on these criteria.”*

5. Line 138-139 mentions a change in protocol halfway through the study (i.e. timing of the occlusion goggles), which due to the non-random group allocations almost overlaps with the separation between experimental and control group. Can the authors discuss whether they think this limitation might have influenced the outcomes?

*The opening and closing of the shutters is especially important in the wash-out phase of the experimental group. Since we do not explicitly compare the experimental and control groups, this change has not affected our outcome.*

6. Line 139 mentions the percentage of trials with occlusion errors. What happened to these trials? Were they deleted and if not, did they lead to outliers in the data?

*Outcomes are the same with and without the trials in which the shutters did not close. Our results report the analysis of all trials. We have added this to the manuscript: “Note that we have included all trials, even those in which the shutters did not close.”*

7. Line 193-195. What was done with the data from the three control participants with vision of the start of their backward movement. If this data was used, were these outliers?

*We included these data. We added the following sentence: “The respective trials were included in the analyses, since they did not systematically deviate from the data of the other participants.”*

8. Line 233-235. Do ‘A’ and ‘B’ here refer to ‘Block A’ and ‘Block B’? Please specify.

*The referee is correct. We have added ‘block’.*
