# Supplementary material for: Single versus dual-rate learning when exposed to Coriolis forces during reaching movements
Source: PLoS One. 2020 Oct 19;15(10):e0240666. doi: 10.1371/journal.pone.0240666 (PMC7571717; doi:10.1371/journal.pone.0240666)
Supplement: Attachment — Submitted filename: Response_to_reviewers.docx [file pone.0240666.s005.docx]

Revision PONE-D-20-15875 R1 “Single versus Dual-Rate Learning when Exposed to Coriolis Forces during Reaching Movements” by Rudolph et al.

**We thank the reviewers for their re-evaluations. Below, you will find the reviewers’ remaining comments, and our reply in bold. We hope that our manuscript is now acceptable for publication.**

Reviewer #1: The authors have adequately responded to my questions. I do have some additional minor comments most of which relate to adding nuance to the conclusion that adaptation is governed by a single-rate process. I think one could alternatively conclude that adaptation is governed by a fast process. In other words: a difference in the system used or in the parameters used.

**We are not sure we completely understand the comment. If we conclude that Coriolis force adaptation is governed by a fast process, that is also a single-rate process.**

1a. What would be the minimal contribution of a slow process that could be detected with the model comparison?

I think it would be interesting to simulate predictions of the two models with expected parameters (taking slow and fast state values from the literature) and with the observed level of noise. Would the dual rate model win the BIC comparison based on the simulated data?

**This is an interesting point but a study on its own; see for example Albert and Shadmehr (2018) to appreciate the complexities involved and assumptions needed to perform such an analysis. For the present study, it requires us to make too many different assumptions, including an estimation of the individual noise levels in the single-rate and dual rate process, the learning and retention rates of the processes, which we feel does not really make an important contribution to our work. Our goal has been to present the data, obtained using a carefully designed paradigm, and perform model fitting to the data using well-established methods. We have been extremely careful, as conveyed by many explicit sentences, to not over-interpret our conclusions - adding further modeling simulations would not allow us to make stronger claims. Note, for the reviewer’s interest, we did run BIC comparisons between single-rate and dual-rate fits on an existing spontaneous recovery dataset from a force field adaptation paradigm, showing evidence for the latter.**

1.b Interpretation of BIC comparison

There might be some nuance to the interpretation of the BIC comparison. When the model with fewer parameters wins, does this mean that this model provides a significantly better account of the data (line 532) or does it mean that adding two parameters did not result in better information?

**We understand this point; we regard the BIC analysis as a maximum likelihood estimate driven method of assessing the model fits penalized for the number of free parameters. Using this analysis, gave us strong to very strong evidence in favor of the single-rate model fits. However, the BIC does not make any statement about the goodness of fit and in theory it could be that both the single- and dual rate model do not fit the data well. That’s the reason that we also present R^2^-values. These values indicate reasonable fits, that do not really differ between the models, as we stated in the revised manuscript. To meet the reviewer’s request of adding more nuance we now changed ‘better account’ into ‘a more parsimonious description” at several places in the MS. Furthermore, we realize that the use of ‘significantly’ in line 532 was ill chosen and have also replaced this by “more parsimonious”.**

1.c Interpretation: single rate versus efficient fast process

In the discussion the authors might provide nuance to adaptation either being governed by a dual- or single-rate process. One could alternatively argue that adaptation is always governed by a multi-rate process in which the contribution of the slow rate depends on how fast the fast state can learn. When the fast state can learn very quickly, there are little errors left to learn from for the slow state.

In this respect, it is interesting that the learning rates for the fast process do not seem very high, for instance compared to my own work (van der Kooij et al., 2015 PLOS). The fact that errors are reduced quickly seems to be due to the high retention rates rather than to the high learning rates. Consistently, the authors find that asymptotic adaptation is complete whereas many studies in the literature report incomplete asymptotic adaptation, which can be explained by incomplete retention.

**We agree that only if the fastest learning process has a high enough retention rate it can fully compensate for the perturbations and reduce error on its own. As a result, slower processes won’t be able to contribute anymore. We do not want to claim that there are no slower processes, but at least they are not prominent enough to show up in our data and analysis. Even proponents of the popular dual-rate model won’t say that there are no slower processes at play, but that in the specific tasks that they investigate they don’t have a significant influence. We feel that we have already provided sufficient nuance, and added our reservations at several places, in particular the discussion. We now make a reference to Van der Kooij et al.’s work when we discuss the learning and retention rates of our single-rate fits (see l. 601-604 )**

2. Prediction on the control group versus experimental group.

I couldn’t find a clear prediction on the expected difference between the control group and experimental group before the presentation of the results. For instance on line 348 it would be helpful to add a prediction for the washout phase.

**Done. We added that we expect no rebound effects in their washout phase (see l. 353-354 )**

3. Line 320: The authors report that 200 model fits were made using randomly selected starting values. How were these 200 fits converged into a single set of fit parameters?

**We added this information (see l. 324). Across the 200 fits, we selected the set of parameters that yielded the smallest mean squared error.**

Reviewer #2: I commend the authors again on a well written study, with my apologies for taking long to complete this review. The revised manuscript reads well and the revisions address most of my concerns. In fact, I have only two minor issues left, which relate to my earlier point that did not come across clearly in the first review. These relate to the protocol for closing the PLATO spectacles

Line 123-126. This sentence does not accurately summarize the spectacle-closing protocol, as it implies that all spectacle-closing was based on contact with the touch screen, but on line 147-150 we learn this is not the case. I would suggest making some mention of these separate protocols here, and/or, to move up the paragraph on line 143-150 to this point so all spectacle-related information is presented together. Furthermore, later in the text, in line 196-204, it is introduced that a different protocol is used in the washout block, so please mention for which blocks this particular protocol is used.

**We have clarified the initially text. We prefer to bring up the whole paragraph on line 143, given that it also contains information unrelated to the spectacles, which needs to be presented first. We hope that the current adjustment made the spectacle-closing protocol sufficiently clear now (see l.125-128).**

Line 125. Fig 1A does not seem to mention the PLATO spectacles, should this be Fig 1C?

**The reviewer is right. We have corrected the referencing.**
